# Validated Assessment of Ejaculatory and Erectile Function After Unilateral “Z”-Shaped Bladder Neck Incision (ZANCLE): A Prospective Study Using the MSHQ-EjD Short Form and IIEF-5

**DOI:** 10.3390/jcm15197699

**Published:** 2026-10-05

**Authors:** Alessandro Calarco, Pietro Morgia, Carlo Maria Scornajenghi, Marianna Paolillo, Pietro Viscuso, Beatrice Filippi, Antonio Tufano

**Affiliations:** Department of Urology, San Carlo di Nancy Hospital, GVM Care and Research, 00165 Rome, Italy

**Keywords:** primary bladder neck obstruction, ejaculatory dysfunction, MSHQ-EjD, IIEF-5, patient-reported outcomes, TUIP

## Abstract

**Background/Objectives**: Preservation of anterograde ejaculation after transurethral incision for primary bladder neck obstruction (PBNO) has traditionally been assessed with unvalidated binary self-report. We prospectively evaluated ejaculatory and erectile function after the ZANCLE (“Z” Anatomical Needle Cut Leads Ejaculation) technique using two validated patient-reported outcome instruments, the Male Sexual Health Questionnaire–Ejaculatory Dysfunction Short Form (MSHQ-EjD-SF) and the International Index of Erectile Function-5 (IIEF-5). **Methods**: Forty consecutive patients undergoing ZANCLE for PBNO at a single institution were prospectively enrolled between June 2024 and July 2025. MSHQ-EjD-SF (ejaculatory function domain, items for force/volume/frequency, independent bother item) and IIEF-5 were administered at baseline and at 1, 6, and 12 months postoperatively, together with the binary anterograde-ejaculation question used in the parent cohort. Voiding parameters (Qmax, IPSS, PVR) are not described here, as they have already been reported for this technique by our group in a previously published series. **Results**: Median age was 41 years (IQR 35–55). The MSHQ-EjD-SF function domain score was unchanged from baseline to 12 months (10.8 ± 1.3 vs. 11.0 ± 1.1, Δ+0.2, *p* = 0.52), while the bother item improved significantly (1.1 ± 0.9 vs. 0.5 ± 0.6, Δ−0.6, *p* = 0.011). IIEF-5 total score remained stable throughout follow-up (20.1 ± 3.0 vs. 20.5 ± 2.8 at 12 months, Δ+0.4, *p* = 0.58). Anterograde ejaculation was preserved by binary self-report in 100% of evaluable patients at every postoperative timepoint. **Conclusions**: Validated instruments confirm, with greater granularity than binary self-report, that the ZANCLE technique preserves ejaculatory function domain scores and erectile function while reducing ejaculation-related bother. These preliminary findings support incorporation of MSHQ-EjD-SF and IIEF-5 into future prospective evaluations of ejaculation-sparing bladder neck surgery.

## 1. Introduction

Men undergoing surgical treatment for primary bladder neck obstruction (PBNO) are considerably younger than the typical patient population treated for benign prostatic hyperplasia (BPH). In this cohort, preservation of antegrade ejaculation is frequently as important to the treatment decision as relief of obstructive symptoms. This concern is not limited to a subset of patients; fertility, sexual self-image, and the impact on partner relationships are commonly discussed during preoperative counselling. A procedure that resolves obstruction but results in loss of antegrade ejaculation is therefore often considered by these patients to represent only a partial treatment success.

PBNO is increasingly recognized as a distinct entity from age-related BPH, particularly among men in their third to fifth decade with low body mass index, small prostate glands, and disproportionately severe symptom scores [1,2]. Diagnostic delay remains common because the clinical picture overlaps with chronic pelvic pain syndromes and overactive bladder, and urodynamic confirmation is not always obtained before treatment is escalated [3,4]. Alpha-blockers remain first-line therapy, yet reported failure rates are considerable and a substantial proportion of men on long-term treatment describe some degree of ejaculatory impairment, an effect that appears particularly pronounced with more uroselective agents [5,6]. Current European Association of Urology guidance reserves bladder neck incision for men with small prostates who fail or decline medical therapy—precisely the population in which preserving ejaculatory function matters most [7].

In our previously published single-surgeon series of 186 patients treated with the ZANCLE technique, with follow-up extended to 24 months, anterograde ejaculation was reported as preserved in 100% of patients at every follow-up visit [8]. That figure is reassuring, but it rests entirely on a single yes/no question asked at each visit—a pragmatic choice, common in the literature, yet one that cannot distinguish a patient with fully normal ejaculation from one who ejaculates with reduced force, volume, or frequency but still answers “yes.” We identified this as a limitation of the parent study and stated explicitly that the next step should be to repeat the assessment using instruments specifically designed and validated to measure ejaculatory function.

Two instruments meet these requirements. The MSHQ-EjD-SF is a four-item, validated questionnaire assessing ejaculatory force, volume, and frequency, plus a separate bother item [9,10]. The IIEF-5 was used as a control measure, to exclude a concomitant effect on erectile function [11]. Both were used together in thulium laser incision of the prostate, a comparable ejaculation-sparing procedure for benign prostatic obstruction. In that cohort, IIEF-5 scores remained stable at two years. Antegrade ejaculation, however, was lost in approximately one in ten patients on binary assessment [12].

Whether this pattern applies to ZANCLE is unknown. We hypothesized that the near-universal preservation of ejaculation previously reported with binary assessment would not fully withstand scrutiny with a validated, multidimensional instrument. We tested this hypothesis in a first cohort of 40 patients undergoing ZANCLE for primary bladder neck obstruction, using the MSHQ-EjD-SF and IIEF-5.

## 2. Materials and Methods

### 2.1. Study Population

This is a single-centre, single-surgeon study. The MSHQ-EjD-SF and IIEF-5 questionnaires were administered prospectively, at defined timepoints, as part of routine perioperative sexual-function counselling; the resulting data were subsequently analyzed retrospectively for research purposes under an Institutional Review Board waiver, as detailed in the Institutional Review Board Statement below. Forty consecutive patients undergoing the ZANCLE procedure for PBNO were identified between June 2024 and July 2025. These 40 patients represent a subset of the previously reported 186-patient ZANCLE series [8]: from a defined point onward during that series’ enrollment, the MSHQ-EjD-SF and IIEF-5 questionnaires began to be administered prospectively, at defined postoperative timepoints, in addition to the standard clinical follow-up already reported for the full parent cohort. The present report describes this validated-instrument sub-cohort specifically. Inclusion and exclusion criteria, diagnostic work-up, and surgical technique were identical to those previously described in that series [8] and are summarized only briefly here. All patients self-reported anterograde ejaculation and intact erectile function at baseline. Written informed consent was obtained from all patients. No a priori sample size or power calculation was performed; the cohort size was determined pragmatically by the number of consecutive patients treated during the study window. Accordingly, the present findings should be regarded as exploratory and hypothesis-generating rather than confirmatory. Because all patients in this sub-cohort had also contributed to the parent series, the present report should be read as a complementary analysis of the same population rather than as an independent replication.

### 2.2. Surgical Technique

All procedures were performed under spinal anesthesia using a monopolar or bipolar resectoscope with a Collins knife, via a unilateral “Z”-shaped incision of the bladder neck and prostate extending toward the verumontanum, with systematic preservation of paracollicular and supramontanal tissue and of approximately 1 cm of prostatic tissue proximal to the verumontanum, as previously described [8]. The unilateral configuration was designed to relieve the functional obstruction at the level of the bladder neck while leaving the contralateral bladder neck and the structures involved in the emission and expulsion phases of ejaculation undisturbed. All procedures were performed by the same surgeon throughout the study period, which minimized technical variability within the cohort.

### 2.3. Sexual Function Assessment

Ejaculatory function was assessed with the MSHQ-EjD-SF, a validated four-item instrument comprising three ejaculatory-function items (force, volume, and frequency of ejaculation, each scored 0–4, domain score range 0–12, higher scores indicating better function) and one independent bother item (scored 0–4, higher scores indicating greater bother) [9,10]. Erectile function was assessed with the IIEF-5, a five-item validated instrument with a total score range of 5–25, higher scores indicating better erectile function [11]. Both instruments, together with the binary anterograde-ejaculation question used in the parent cohort, were administered at baseline and at 1, 6, and 12 months postoperatively. Voiding parameters (Qmax, PVR, IPSS, IPSS-QoL) were not re-analyzed in the present report and are reported in the previously published series [8]. The MSHQ-EjD-SF was selected because it measures the physical components of ejaculation separately from the distress associated with them, whereas the IIEF-5 was included to detect any concomitant change in erectile function that could confound the interpretation of ejaculatory outcomes. Questionnaires were self-administered at each scheduled visit.

### 2.4. Statistical Analysis

Continuous variables were summarized as medians with interquartile ranges (IQR) or as means ± standard deviation, as appropriate; normality was assessed with the Shapiro–Wilk test. Within-patient change from baseline (Δ) was calculated for MSHQ-EjD-SF and IIEF-5 scores at each follow-up timepoint (T1 = 1 month, T2 = 6 months, T3 = 12 months vs. T0 = baseline) and compared using the Wilcoxon signed-rank test, consistent with the analytic approach used for ΔIPSS/ΔIIEF-5 reporting in comparable ejaculation-sparing series [12]. Categorical variables were compared with the chi-square or Fisher’s exact test. Spearman’s rho was used to explore the correlation between ΔMSHQ-EjD bother and ΔIIEF-5. Statistical significance was set at *p* < 0.05. Analyses were performed using SPSS v.29.0 (IBM Corp., Armonk, NY, USA). Changes across all four timepoints were additionally assessed with the Friedman test. For each score, the mean within-patient change at 12 months was reported together with its 95% confidence interval (95%CI). The proportion of patients with preserved anterograde ejaculation was reported with an exact (Clopper–Pearson) 95%CI, which is appropriate when no events are observed. As all patients completed the 12-month visit, no imputation of missing data was required.

## 3. Results

Forty patients met inclusion criteria and completed baseline assessment; all patients completed the scheduled 12-month visit. Baseline and perioperative characteristics are summarized in Table 1.

MSHQ-EjD-SF and IIEF-5 scores at each timepoint, with within-patient change from baseline, are reported in Table 2. The MSHQ-EjD ejaculatory function domain score did not change significantly over the study period (Friedman *p* = 0.47; Δat 12 months +0.2, 95%CI −0.3 to +0.7, *p* = 0.52). The MSHQ-EjD bother item improved significantly from baseline, with a progressive reduction sustained at 12 months (Δ−0.6, 95%CI −1.1 to −0.1, *p* = 0.011). IIEF-5 total score remained stable across all timepoints (Δat 12 months +0.4, 95%CI −0.9 to +1.7, *p* = 0.58), indicating no clinically relevant change in erectile function. Anterograde ejaculation was preserved by binary self-report in 100% of evaluable patients at every postoperative timepoint, with no cases of new-onset ejaculatory dysfunction recorded through 12 months. Given the absence of events, the exact (Clopper–Pearson) 95% confidence interval for the observed 100% (40/40) preservation rate was 91.2% to 100%; this interval, rather than the point estimate alone, should be used to interpret the generalizability of this finding.

Table 3 reports within-patient Δvalues (median, IQR) for MSHQ-EjD-SF and IIEF-5 at each follow-up timepoint versus baseline.

Upon exploratory analysis, ΔMSHQ-EjD bother and ΔIIEF-5 at 12 months were not significantly correlated (Spearman’s rho = 0.14, *p* = 0.39), suggesting that improvement in ejaculation-related bother was largely independent of erectile function status. No ejaculatory events occurred in this cohort, precluding regression analysis of predictors of ejaculatory loss; such analysis would require a larger series with an adequate number of events.

## 4. Discussion

Voiding parameters are not reported in this study. They were previously established, with durable improvement to 24 months, in our 186-patient series [8]. The present study was designed to address a different endpoint, namely validated sexual function, rather than to repeat that analysis. Keeping the two endpoints separate also avoided duplication of previously published data and allowed the present analysis to focus exclusively on sexual function.

This is the first prospective assessment of ejaculatory and erectile function after ZANCLE using validated instruments rather than binary self-report alone. It directly addresses a limitation identified in the parent series [8]. By applying validated instruments to a consecutive cohort with complete follow-up, the present study provides a more detailed characterization of sexual function after ZANCLE than was previously available.

The MSHQ-EjD function domain remained unchanged, from 10.8 ± 1.3 at baseline to 11.0 ± 1.1 at 12 months (*p* = 0.52). The bother item improved significantly, from 1.1 ± 0.9 to 0.5 ± 0.6 (*p* = 0.011), with the reduction already evident at 6 months (*p* = 0.02) and sustained thereafter. IIEF-5 remained stable, from 20.1 ± 3.0 to 20.5 ± 2.8 (*p* = 0.58). Change in bother and change in IIEF-5 did not correlate (rho = 0.14, *p* = 0.39), indicating that the reduction in ejaculation-related bother was not attributable to erectile function.

Our hypothesis was only partially confirmed. We anticipated that a validated instrument would reveal impairment not captured by binary self-report; this hypothesis was not supported by our data. The function domain remained unchanged across follow-up, and binary preservation of ejaculatory function was maintained in 100% of patients throughout the study period. Rather than correcting the binary outcome, the validated instrument identified a distinct dimension—ejaculation-related bother—that the binary question was not designed to capture.

This finding admits two interpretations. Either the binary question used in the parent series was already an adequate proxy for ejaculatory function in this specific population, or the sample size was insufficient to detect small degrees of impairment. The high baseline scores and the narrow distribution of within-patient changes favour the first interpretation, although the second cannot be excluded.

Function and bother do not necessarily move together. A patient may report unchanged force, volume, and frequency of ejaculation, yet experience it differently once the straining and interrupted flow that prompted surgery have resolved. In this cohort, bother, rather than the function domain, was the more informative outcome.

Several mechanisms may explain this dissociation. Before surgery, most patients (72.5%) were receiving alpha-blockers, a drug class that can impair ejaculation, particularly in its more uroselective forms [5,6]. Relief of the obstruction may have allowed medical therapy to be reduced or withdrawn, with a consequent improvement in the perceived quality of ejaculation even in the absence of measurable change in force, volume, or frequency. In addition, men with PBNO may experience straining, perineal discomfort, and a sense of incomplete emptying, which can influence the perception of sexual function as a whole. Once these symptoms subside, ejaculation may be experienced as less troublesome, even if its physical characteristics remain unchanged. The present analysis did not account for postoperative changes in medical therapy; these explanations should therefore be regarded as hypotheses rather than conclusions.

The absence of change in the function domain should also be interpreted in light of baseline values. Patients started from a high score (10.8 of a maximum of 12), which leaves little room for improvement and, conversely, makes a small decline more difficult to detect. This ceiling effect is inherent to a population of young men who reported anterograde ejaculation before surgery, and it implies that, in this setting, the MSHQ-EjD-SF is better suited for identifying deterioration than for demonstrating benefit. Moreover, no minimal clinically important difference was predefined for this instrument in the present study. The small and non-significant changes observed in the function domain and in the IIEF-5 are therefore unlikely to be clinically meaningful, but their precise relevance cannot be quantified.

Erectile function remained stable throughout follow-up. Mean IIEF-5 scores at baseline and at 12 months fell within the same category of the instrument [11], and the median within-patient change was zero at every timepoint. This finding is relevant for counselling, because concern about erectile function is frequently raised by young men considering endoscopic surgery, even though the bladder neck incision is performed at a distance from the cavernous nerves.

Transurethral incision of the bladder neck has long been the surgical reference for PBNO, with favourable treatment results reported in early urodynamic series [13]. However, disturbances of ejaculation remain the main drawback of transurethral procedures and weigh heavily on quality of life in younger men [14,15]. Endoscopic incision has also proved effective in men with previously unrecognized vesical neck obstruction, underlining the importance of accurate diagnosis before surgery [16]. Building on the initial single-surgeon experience with ZANCLE [17], several other ejaculation-sparing strategies have been described, including modified and unilateral bladder neck incisions [18,19], ejaculatory hood-sparing vaporization [20], and minimally invasive devices that avoid tissue ablation [21].

These results are consistent with comparable series. In thulium laser incision of the prostate, IIEF-5 was stable at 2 years, while binary preservation of antegrade ejaculation was 88% (14 of 120 patients with loss) [12]. That cohort was older (median 55 versus 41 years) and had larger prostates (35 versus 26 mL); age, prostate volume greater than 30 mL, median lobe, and operative time were independent predictors of ejaculatory loss on regression analysis [12]. In diode laser enucleation using the Leonardi technique, antegrade ejaculation was preserved in 94.1% of patients, with IIEF-5 improving from 14.4 to 16.2 [22]. Across these three series, anatomical preservation of the paracollicular and supramontanal tissue, rather than the energy source, appears to be the principal determinant of ejaculatory outcome.

The 100% preservation rate observed here, in a younger population with smaller prostates and a smaller unilateral incision, is consistent with a more favourable substrate for ejaculatory preservation than in older BPO populations undergoing more extensive bladder neck ablation. This hypothesis requires direct comparative testing.

It should be emphasized that the absence of events does not equate to the absence of risk. The exact 95%CI for the observed preservation rate extends down to 91.2%, meaning that a true loss rate of up to approximately 9% cannot be excluded on the basis of 40 patients. This lower bound is not far from the rate of ejaculatory loss reported after thulium laser incision [12], and larger samples will be required before any superiority of ZANCLE over other ejaculation-sparing approaches can be claimed.

No ejaculatory loss occurred in this cohort. A predictor analysis comparable to that performed in the ThuIP series [12] could therefore not be undertaken; with no events, no regression model can be constructed. This will become feasible once outcomes are pooled with the parent cohort or with future series in which some degree of event heterogeneity is expected.

From a clinical perspective, these findings have practical implications for preoperative counselling. Young men considering surgery for PBNO can be informed not only that antegrade ejaculation was preserved in all patients in this series, but also that the perceived quality of ejaculation did not deteriorate and that ejaculation-related bother tended to improve. This information is more nuanced than a binary statement and addresses the concerns most frequently raised by this population. From a methodological perspective, our data support the systematic use of validated, multidimensional instruments in future studies of ejaculation-sparing surgery. Binary self-report, although simple and widely adopted, provides limited information and does not allow meaningful comparison across techniques. The adoption of a common set of patient-reported outcomes, including both function and bother, would facilitate pooling of data from different series and allow a more reliable comparison of ejaculation-sparing procedures.

This study has several limitations. The sample size is modest (*n* = 40), and no a priori sample size or power calculation was performed; as noted in the Materials and Methods section, the cohort size was determined pragmatically rather than derived from a predefined minimal clinically important difference, and the study should be interpreted as exploratory rather than confirmatory. The design is single-arm and single-centre, without a comparator technique or energy source, so the present data cannot determine whether the observed preservation of ejaculatory function is attributable specifically to the Z-shaped configuration, to the unilateral approach more generally, to preservation of paracollicular and supramontanal tissue, or to other technical or patient-related factors; a direct comparison with a conventional unilateral bladder neck incision would be needed to isolate this contribution. Follow-up was limited to 12 months, shorter than the 24-month horizon of both the parent ZANCLE cohort and the ThuIP series [8,12]; whether stability persists, or late ejaculatory changes emerge, cannot be determined from the present data. This shorter horizon reflects a deliberate design choice, made to report the first validated-instrument assessment of this cohort at a clinically meaningful early timepoint without delay, rather than an oversight; extension of validated follow-up to 24 months, to match the parent cohort, is planned as a separate, subsequent analysis. Semen volume and other objective andrological parameters were not measured, so preserved ejaculation reflects patient-reported experience rather than laboratory confirmation. Recall and social-desirability bias inherent to self-administered questionnaires cannot be excluded, and the absence of partner-reported or physician-independent corroboration remains a limitation of the literature generally, not only of the present study.

Future research should address these issues in several ways. First, extension of validated follow-up to 24 months will clarify whether the stability observed at 12 months is maintained over time. Second, pooling the present sub-cohort with the remainder of the parent series, and with other centres adopting the same assessment protocol, would provide a sufficient number of events to explore predictors of ejaculatory loss. Third, a prospective comparison between ZANCLE and conventional bladder neck incision, ideally randomized, would be required to establish whether the Z-shaped configuration confers a specific advantage, given that comparative evidence on the surgical management of PBNO remains limited [23]. Finally, the inclusion of objective andrological parameters, such as semen volume, and of partner-reported outcomes would provide a more complete picture of sexual and reproductive function after surgery.

## 5. Conclusions

In this cohort of 40 patients treated between June 2024 and July 2025, validated assessment with MSHQ-EjD-SF and IIEF-5 confirmed complete (100%) preservation of anterograde ejaculation and stable ejaculatory function domain scores and erectile function after ZANCLE, with a significant reduction in ejaculation-related bother, over 12 months of follow-up. These findings indicate that no patient-reported loss of antegrade ejaculation was observed after unilateral Z-shaped bladder neck incision during the study period; they do not, however, establish whether this outcome is specific to the Z-shaped configuration itself or whether comparable results could be achieved with other unilateral bladder neck incision techniques, a question that will require direct comparative study.

## Figures and Tables

**Table 1:** Baseline and perioperative characteristics (*n* = 40).

Variable	*n* = 40
Age at surgery, years, median (IQR)	41 (35–55)
Prostate volume, mL, median (IQR)	26 (19–29)
ASA score 1–2, *n* (%)	37 (92.5)
ASA score 3–5, *n* (%)	3 (7.5)
Preoperative alpha-blocker therapy, *n* (%)	29 (72.5)
Preoperative PSA, ng/mL, median (IQR)	1.5 (0.8–2.2)
Baseline MSHQ-EjD function domain, mean ± SD	10.8 ± 1.3
Baseline MSHQ-EjD bother item, mean ± SD	1.1 ± 0.9
Baseline IIEF-5, mean ± SD	20.1 ± 3.0
Operative time, min, median (IQR)	19 (15–25)
Catheterization time, days, median (IQR)	3 (2–4)
Clavien 1–2 complications, *n* (%)	1 (2.5)

**Table 2:** MSHQ-EjD-SF and IIEF-5 scores at baseline and follow-up.

Variable	Baseline	1 mo	6 mo	12 mo
MSHQ-EjD function domain (0–12)	10.8 ± 1.3	10.5 ± 1.5	10.9 ± 1.2	11.0 ± 1.1
MSHQ-EjD bother item (0–4)	1.1 ± 0.9	0.9 ± 0.8	0.6 ± 0.7	0.5 ± 0.6
IIEF-5 total (5–25)	20.1 ± 3.0	19.8 ± 3.2	20.3 ± 2.9	20.5 ± 2.8
Anterograde ejaculation preserved, *n* (%) *	40 (100)	40 (100)	40 (100)	40 (100)

* *p*-values (vs. baseline, Wilcoxon signed-rank test, 12-month timepoint).

**Table 3:** Within-patient change (Δ) from baseline in MSHQ-EjD-SF and IIEF-5.

Variable	ΔT2–T0 (6 mo)	*p*	ΔT3–T0 (12 mo)	*p*
ΔMSHQ-EjD function domain, median (IQR)	0 (−1; +1)	0.61	0 (−1; +1)	0.52
ΔMSHQ-EjD bother item, median (IQR)	−1 (−1; 0)	0.02	−1 (−1; 0)	0.011
ΔIIEF-5, median (IQR)	0 (−1; +1)	0.58	0 (−1; +1)	0.58

## Data Availability

The data presented in this study are available upon request from the corresponding author. The data are not publicly available due to institutional data-protection policies governing patient-level clinical information.

## Abbreviations

The following abbreviations are used in this manuscript:

PBNO	Primary bladder neck obstruction
BPH	Benign prostatic hyperplasia
ZANCLE	“Z” Anatomical Needle Cut Leads Ejaculation
MSHQ-EjD-SF	Male Sexual Health Questionnaire–Ejaculatory Dysfunction Short Form
IIEF-5	International Index of Erectile Function-5
TUIP	Transurethral incision of the prostate
ThuIP	Thulium laser incision of the prostate
Qmax	Maximum urinary flow rate
PVR	Post-void residual urine volume
IPSS	International Prostate Symptom Score
ASA	American Society of Anesthesiologists (physical status classification)
PSA	Prostate-specific antigen
IQR	Interquartile range
SD	Standard deviation
CI	Confidence interval
